# First Evaluation of a Novel Live-Chat Service for Preventing Child Sexual Abuse

**DOI:** 10.5964/sotrap.15145

**Published:** 2026-09-23

**Authors:** Tom Gregg, Natasha Kalebic, Joshua W. Moore, Georgia Naldrett, Sarah Edwards, Donald Findlater, Pamela J. Taylor

**Affiliations:** 1 School of Medicine, Cardiff University, Cardiff, United Kingdom; 2 School of Psychology, Cardiff University, Cardiff, United Kingdom; 3 Oxleas NHS Foundation Trust, London, United Kingdom; 4 Wolfson Centre for Mathematical Biology, Mathematical Institute, University of Oxford, Oxford, United Kingdom; 5 Lucy Faithfull Foundation, Bromsgrove, United Kingdom; Centre for Criminology (Kriminologische Zentralstelle – KrimZ), Wiesbaden, Germany

**Keywords:** helplines, chatlines, online messaging services, child sexual abuse prevention, Stop It Now!

## Abstract

Live-chat services are increasingly used for prevention in healthcare. Systematic literature searching found them untested for preventing sexual abuse of children. Our aims were to compare anonymous cohorts using a new live-texting service *or* an established phoneline with the same prevention aims and drawing on the same responder pool, and explore chat qualities. In the first three months of live-chat, all 120 hours available were used. Chatters (*n* = 104) were significantly younger than phoneline-users (*n* = 985), over half of both groups help-seeking for their own inappropriate activities. Significantly fewer live-chat (49%) than phoneline-users (82%) reported any police involvement. Qualitative analysis of self-concerned chat transcripts suggested that, while psychological engagement with responders seemed unusual, 10%, returned on subsequent days and carried ideas between chats. Their core concern was of internal ‘struggle’, especially around self-identity, rendering some suicidal. In reaching undetected perpetrators, this first evaluation of live-chat in this context suggests greater primary preventive potential than the phoneline. As psychological engagement seems feasible, next steps should include developing engagement strategies.

## Abstract (plain language summary)

**Background:**

Sexual activity towards children is likely to inflict lasting harms. Even if behaviour has been confined to illegal use of images, real children may have been harmed when those images were made. Optimal prevention is ‘primary prevention’, when someone may be thinking of such actions but has not yet done anything. This is hard to achieve because open help-seeking may precipitate criminal justice system involvement and/or such strict safeguarding measures that families are broken up. Anonymous telephone helplines have been operative for some years to try to prevent new or repeated offending. New live-chat options are being introduced.

**Why Was This Study Done?:**

Any new service needs evaluation. If not achieving what they set out to do, or not adding anything, they need redesign or closure. When preliminary findings are positive, they will require research-informed adjustment to support best outcomes.

**What Did the Researchers Do and Find?:**

We compared users of a new live-chat service with users of a well-established phone-helpline delivered by the same organisation, using anonymous records. We also examined chat transcripts to see how chatters concerned about their own behaviours worked with the responding advisors. We found that live-chat users were younger than phoneline users and less likely to have had any contact with the police. Many self-concerned live-chat users made only one contact, but 10% returned and showed evidence of using the opportunity to start changing.

**What Do These Findings Mean?:**

These findings suggest that the live-chat service reaches people earlier than the phoneline and, therefore, offers more opportunity for primary prevention. Exploration of the self-concerned chatters suggests at least some have a capacity for change, so work to enhance engagement in the process of change would be worthwhile.

## Highlights

- Anonymous helplines are a vital resource for taboo behaviours, such as sexual interest in children.
- Helplines for child sexual abuse prevention are established, but such use of live-chat untested.
- Records comparison of phone and live-chat users found live-chat reached more people unknown to police.
- Most live-chatters made one contact, but 1/10 showed evidence of engagement in psychological work.

Telephone helplines for preventing harms by people concerned about their own behaviours have been operating for decades. In 1953, for example, Chad Varah established the Samaritans’ helpline ‘just willing to listen’ to people with suicidal thoughts. Developments in digital technology have meant increasing availability of live-chat services. Towards the end of 2020, the UK-based Lucy Faithfull Foundation (LFF), set up to prevent sexually inappropriate or abusive behaviours involving children, offered a new service, responding to texts from people with such concerns.

The mix of working remotely and, usually, anonymously, with people in distress makes evaluation of prevention effectiveness very difficult. A systematic review of studies of prevention of perpetration of child sexual abuse located only full treatment interventions; of the seven included, just one was online (Seto et al., 2024). Hoffberg et al. (2020) conducted a systematic literature review of studies of crisis/help line effectiveness, including but not confined to live-chat, but none for sexual behaviours. They considered that little weight could be put on outcomes, because of the immediacy of measures and reliance on self-rating, the latter often just as ‘satisfaction’ after the call. They called for ‘innovative strategies … with a specific focus on … engagement’ (here behavioural health treatment engagement).

In the wider health promotion field, Brody et al. (2020) completed a systematic review of chat-based hotlines specifically, with only two of twelve included papers overlapping with the Hoffberg group’s review. The twelve were of services offering 1. free and confidential opportunities, 2. helping those contacting to identify and understand the behaviours they need to change and 3. to set goals to achieve this, partly informed by responders. Most were for physical health promotion, but four focussed on behavioural problems, of which one related to sexually inappropriate behaviours, albeit exclusively sexual assault survivors. Again, limits on outcome data were acknowledged, the main finding being that users were positive about the services. In a ‘synthesis of contemporary literature’ on mobile technology to support health for older adults, Kuerbis et al. (2017) found that most of those physically and mentally able to use mobile phones were willing to engage with live-chat, with ease of accessibility and attractiveness of design influencing use. Outside health, in a review of library-supported live-chat, Matteson et al. (2011) found that convenience of access was the main variable affecting use. Live-chat users liked its informality and immediacy.

We conducted our own systematic search for literature up to 26/11/2023 on chat/text-based interventions for intimate abusive behaviours, encompassing a broader field than child sexual abuse alone in order to maximise chances of finding relevant material. Terms for live-chat messaging AND terms for abusive behaviours were entered into each of seven databases: Web of Science, PsycInfo, Medline, ASSIA, CINAHL, ERIC and SCOPUS (all search algorithms are presented in full in the Online Supplement 1). Five hundred and four unique titles were returned, of which 472 could be excluded on titles and abstracts and the remaining 32 papers after reading full text. None related to live-chat/texting *and* child sexual abuse, although we did find an ongoing University of Texas evaluation of chat hotlines established in Texas for ‘victims of sexual assault and exploitation, intimate partner violence, human trafficking, and child abuse and neglect’, confirming user satisfaction; we can trace no updates since 2023 (https://nij.ojp.gov/topics/articles/evaluating-victim-services-text-and-chat-hotlines-technology-helps-reach-underserved-populations).

In this context of so few data on live-chat services for prevention of harms, and none about actual or potential perpetrators of child sexual abuse, our aim was to explore use of the new LFF live-chat option for preventing child sexual abuse. Our first question was about the extent to which the service would be used, our second about whether and how live-chat users differ, if at all, from conventional phoneline users – and, thus, whether live-chat could be reaching a new clientele. Our third question was about how chatters used the service and any evidence of meaningful engagement with responders by two measures: whether they returned for further live-chats and in terms of qualities in expression of their concerns.

## Method

### Ethics

Approval for the study was from the LFF ethics committee, the organisation hosting the chatline. As this work fitted criteria for a service evaluation and used anonymised data, no further approvals were required (http://www.hra-decisiontools.org.uk/research/index.html).

### Research Design

We used mixed methods, quantifying chat-line users and their characteristics in terms of age, sex, nature of stated problem and police involvement, and using a modified grounded theory approach (Glaser & Strauss, 1967; Glaser 1998) to analyse chat transcripts.

### The Stop It Now Chat-Line

In 2002, the Lucy Faithful Foundation (LFF) established the Stop it Now UK and Ireland telephone helpline ‘to make sure adults know what they can do to keep children safe’ – specifically from sexual abuse. In late September 2020, the Foundation first implemented a live-chat/texting feature. Stop It Now services in the USA and Belgium (Flanders) have also introduced live-chat, but not evaluated it so far.

The live-chat service is provided by responders with relevant health, psychology, social work or criminal justice background, specific helpline training and additional training for live-chat responding. Its introduction was not widely advertised but explicitly offered on The Foundation’s website. At time of this research, as a pilot project, it was available for three four-hour periods per week.

Every new contact with a Stop It Now responder is given a unique personal number and each contact a unique contact number, using a *Salesforce* operating system proforma. Initially, people who contact the service are asked for a made-up first name and a true county of residence, the latter to facilitate advice on availability of services. For added anonymity, at this stage everyone is also asked for an ‘age bracket’ (see Online Supplement 2) rather than specify age or date of birth. In the event of further contact, the three datapoints of unique LFF number, chosen name and county are used to check the contactor identity. Callers might make new calls using different identities, but responders always ask about prior contact and try to link files, and review cases together periodically. There have been rare occasions when the story of a purportedly new caller suggests otherwise. All those designated as repeat callers/chatters in our study have had records linked as described.

The Foundation provides other forms of support and interventions, not all anonymously, but this study is confined to the anonymous live-chat and phoneline services.

### The Sample

Records of all new ‘chatters’ between 28^th^ September and 31^st^ December 2020 and all new phone-callers for the same period 24 months previously formed the comparison cohorts. The comparison caller period was chosen to ensure that none in the comparison group was also using the chatline and because call types may be season related. In view of 2020 pandemic-related effects, we also checked patterns of conventional phone call type by sex, age, call type and reported police involvement of phoneline users between 2018 and 2020. The total number of new calls for 2020 was higher (1,130) than in 2018 (987), but all other parameters similar: mean age of callers (2018: 41.65 [*SD* = 14.27]; 2020: 42.72 [*SD* = 14.20]; 60% of each cohort were men. Problem type distribution was similar, with around 54% in both concerned about their own behaviours, and 78% (2018) and 72% (2020) reporting police involvement.

### Data Extraction

Data on number of contacts, sex, age, reason for/nature of contact and police involvement were extracted into a database from the records according to a data extraction sheet (see Online Supplement 2). All calls and chats are anonymous, but we further anonymised by applying a research number to replace the Salesforce number.

Full transcripts of the live chats were obtained and all given names and geographic location data removed. For these purposes, single occasion chatters were those who had made a first single contact on one day or the repeat contact had been on that day to restore a dropped line. Definition of multiple time chatters was that at least two chats had occurred on at least two separate days and the repeated contact was not just about simple practicalities such as confirmation of receipt of advice or an appointment. Given that we adopted qualitative methods here, there was no formal comparison between single and multiple occasion chatters; we analysed their data separately.

### Analyses

#### Quantitative Data

The complete samples of chatline users and phone-callers were compared by descriptive statistics, using SPSS V23. Nonparametric analysis of age band distributions was performed using the Statistics and Machine Learning Toolbox in Matlab (v2021a) (Bowman & Azzalini, 1997). Multivariate analyses were conducted to explore direction of relationships significant in bivariate analyses for the group concerned about their own behaviours.

#### Narrative Data

As we had no preconceived ideas about the nature of the chats, we used a modified grounded theory approach to qualitative analysis (Glaser & Strauss, 1967; Glaser 1998); modification was necessary because each narrative contained some material more-or-less directed by the responder. Key interventions by the chat-responder and responses to direct questions were noted, but not included as data categories.

The first narrative was examined, and categories of spontaneous text extracted into a table, where possible labelling each category with a word or phrase used by the chatter; the full quotation was the supporting evidence. For multiple occasion live-chats, the order of the chat was also noted. The second narrative was analysed in a similar way, using already identified categories where possible and adding new ones as necessary. Two of us analysed the first three narratives, blind to each other. We found small differences in the extent to which each item was listed as a separate category – for example both of us identified ‘monstrous’ and ‘self-hate’ as extreme reactions, but only one named ‘absolutionist thinking’ as a separate first order category. We agreed that all these fitted with a higher order category of ‘extreme self-denigration’, but nevertheless to initial listing of every possible first order category. Blind analyses of three further cases showed full agreement. Remaining extractions were completed by just one of us for each set: 1) exclusive chatline users initiating more than one live-chat (PJT) and 2) single-occasion chatters (TG).

Using constant comparative analysis, higher order categories were allowed to emerge from similarities and differences between first order categories, then the transcripts re-read applying these categories to check their fit. Finally, a ‘core category’ which best encompassed all the other categories was sought.

## Results

### Extent of Live-Chat Service Use

In the 95 days between the live-chat launch on September 28^th^ and census date of 31^st^ December 2020, staff made 120 hours available. All these hours were used. 143 different ‘clients’ used live-chat at least once, most (105, 73%) using this alone; 38 also used the phoneline. Eighty-eight (84%) of sole live-chat users did so once only, nine on two occasions at least 24 hours apart and seven on three or more separate days. A further four connected for multiple chats within one day, but not otherwise during the study period. The largest number of chats by one service user on separate days during the period was 17.

Two-thirds of people using live-chat were men (overall: 95, 66% men; 44, 31% women; 4 not known [NK]; proportions identical for exclusive live-chat users). Mean [*M*] age was 35.41 years (*SD* = 13.38, range 14–65 years). No-one over 65 used live-chat; the line is offered for adults only but, three under-18s made contact.

Just over half (55, 52%) of people who used live-chat alone were concerned about their own behaviour, all but six of these identifying as men (3 women, 3 NK). Twenty-nine (28%) were concerned about the behaviour of someone close to them, e.g. partners, ex-partners, parents, all but 8 of these identifying as women. A miscellaneous group of 20 chats included professionals seeking advice.

#### People Who Used Both Forms of Contact

Among the 38 people using both systems, there was no apparent preference for first contact (20 first live-chat: 18 first phoneline); 16 used the options about equally, although often using the phone to talk and live-chat for getting service information; among the other 22, just three relied more on live-chat. About a third (11), had turned to live-chat only after failing to access the phoneline.

### Comparison of Live-Chat and Conventional Phoneline Users

Table 1 shows details of the age band distribution of the pure live-chat users, pure phoneline users and the mixed group. These confirmed that the age difference between service type users was mainly accounted for by about twice the proportion of under-30s preferring live-chat to the phoneline. Under 7% of chatters were over 60 and none over 65; just over 10% of phoneline users were over 60, with nearly 3% aged 70+.

**Table 1:** Age Distribution of Live-Chat Users Compared With Conventional Phoneline Users

Type of Helpline Use	Age in Years												Total with data		Missing data		Totals
	< 18		18-29		30-39		40-49		50-59		> 59						
	*n*	%	*n*	%	*n*	%	*n*	%	*n*	%	*n*	%	*n*	%	*n*	%	*N*
Live-chat	5	4.8	30	28.6	32	30.5	15	4.3	12	11.4	7	6.7	101	96.2	4	3.8	105
Conventional phoneline	7	0.7	137	14.0	209	21.2	180	18.2	150	15.2	102	10.3	785	79.2	202	20.5	987
Mixed live-chat and phone use	1	2.6	12	31.6	10	26.3	5	13.2	7	18.4	2	5.3	37	97.4	1	2.6	38

*Note.* Age distribution data are from age band estimates, which include but are not confined to information about exact age if the caller prefers less precise disclosure. Mean [*M*] age group differences by communication preference: users of live-chat only *M* 34.14 years, *SD* 13.3, range 14-65; exclusive phoneline users *M* 42.72, *SD* 14.2, range 12-88, *t*(830) = 4.115, *p* < .01; mixed users *M* 37.24, *SD* 13.48, range 17-63. Mean age difference by communication preference in the two largest groups of users – those concerned about own behaviours and those concerned about the behaviour of other: self-concerned chatters *M* 35.18, *SD* 13.2: phone users *M* 41.84, 14.62, *t* = (545) 3.234, *p* < .01; other-concerned chatters *M* 36.82, *SD* 11.43: phone users 45.83, *SD* 12.85, *t* = (260) 3.176, *p* < .01.

Overall, those exclusively using live-chat were significantly younger. Subgroup checks confirmed that the difference in age distribution between those concerned about their own behaviours and those people concerned about ‘significant others’ were each, separately, in line with this (see Table 1).

Men and women were about as likely to use live-chat as the voice service (chatline only 68 men, 67%; phoneline only 596 men, 62%; both 27, 71%; u = 49675.5, *p* = .837). Table 2 shows that the distribution of main type of concern was similar between pure live-chat and pure phoneline users. Distributions were more varied among people using both but, as some of these groups were so small, they were excluded from analyses.

**Table 2:** Comparison of Live-Chat and Phoneline Users by Type of Concern

Type of Helpline Use	Concern Type						‘Bystanders’^a^		Others^b^		Victims / survivors		Totals
	Self-concern about												
	contact abuse		online abuse		accessing illegal images								
	*n*	%	*n*	%	*n*	%	*n*	%	*n*	%	*n*	%	*N*
Live-chat users	17	16.3	7	6.7	31	29.8	33	31.7	13	12.5	3	2.9	104
Conventional phone users	122	12.4	59	6.0	328	33.3	333	33.8	126	12.8	16	1.6	985
Mixed live-chat & phone users	6	15.8	8	21.1	8	21.1	14	36.8	2	5.3	0	0.0	38

*Note.* The relationship between pure live-chat use vs pure phoneline use by type of concern not significant: $\chi_{5}^{2}$ = 2.57, *p* = .766.

**^a^**‘Bystanders’ is the term used to describe anyone who is related to or in contact with the abuser/online offender/suspected abuser or online offender or a victim but not themselves abusing or experiencing direct personal abuse. **^b^**4 professionals seeking advice, 9 general information requests; 1 ‘inappropriate caller’.

Examination of any caller-reported police contact showed that pure live-chat users concerned about others (excepting professionals, survivors, and information requesters) were less likely to report police involvement than pure phoneline users (Table 3a).

**Table 3a:** Police Involvement Status Where Concerns Were Brought by Others (‘Bystanders’)

Service used	No involvement		Police involvement		Missing data		Totals^a^
	*n*	%	*n*	%	*n*	%	*N*
Live-chat	15	45.5	15	45.5	3	9.1	33
Conventional phone	72	21.6	243	73.0	18	5.4	333
Mixed live-chat and phone	3	21.4	10	71.4	1	7.1	14

*Note.* Police involvement refers to any reported contact with police specifically about sexual activity relating to children, including accessing illegal images of children online. This may or may not have resulted in criminal charges and may or may not refer to the immediate reason for the call. Excluding the mixed service use group: $\chi_{1}^{2}$ = 10.701, *p* < .01.

^a^164 cases not included in these analyses because they were professionals seeking advice, requests for general information, survivor related calls or inappropriate callers; missing data noted and excluded.

Among the 55 chatline and 509 conventional phone users concerned about their own behaviour, significantly fewer pure live-chat than pure phone users reported any police involvement prior to the first chat (Table 3b).

**Table 3b:** Police Involvement Status Among Those Reporting Concerns About Their Own Behaviour

Service used	No police involvement		Police involvement		Missing data		Totals
	*n*	%	*n*	%	*n*	%	*N*
Chatline users	23	41.8	27	49.1	5	9.1	55
Conventional phone users	88	17.3	416	81.7	5	1.0	509
Mixed chatline^a^ and phone users	5	22.7	16	72.7	1	4.5	22

*Note.* Excluding the mixed service use group: $\chi_{1}^{2}$ = 23.125, *p* < .01.

^a^The small group of mixed chat and phoneline users concerned about their own behaviour were not included in statistical analyses, but seemed likely to be in an intermediate position with respect to police involvement.

**Figure 1:**
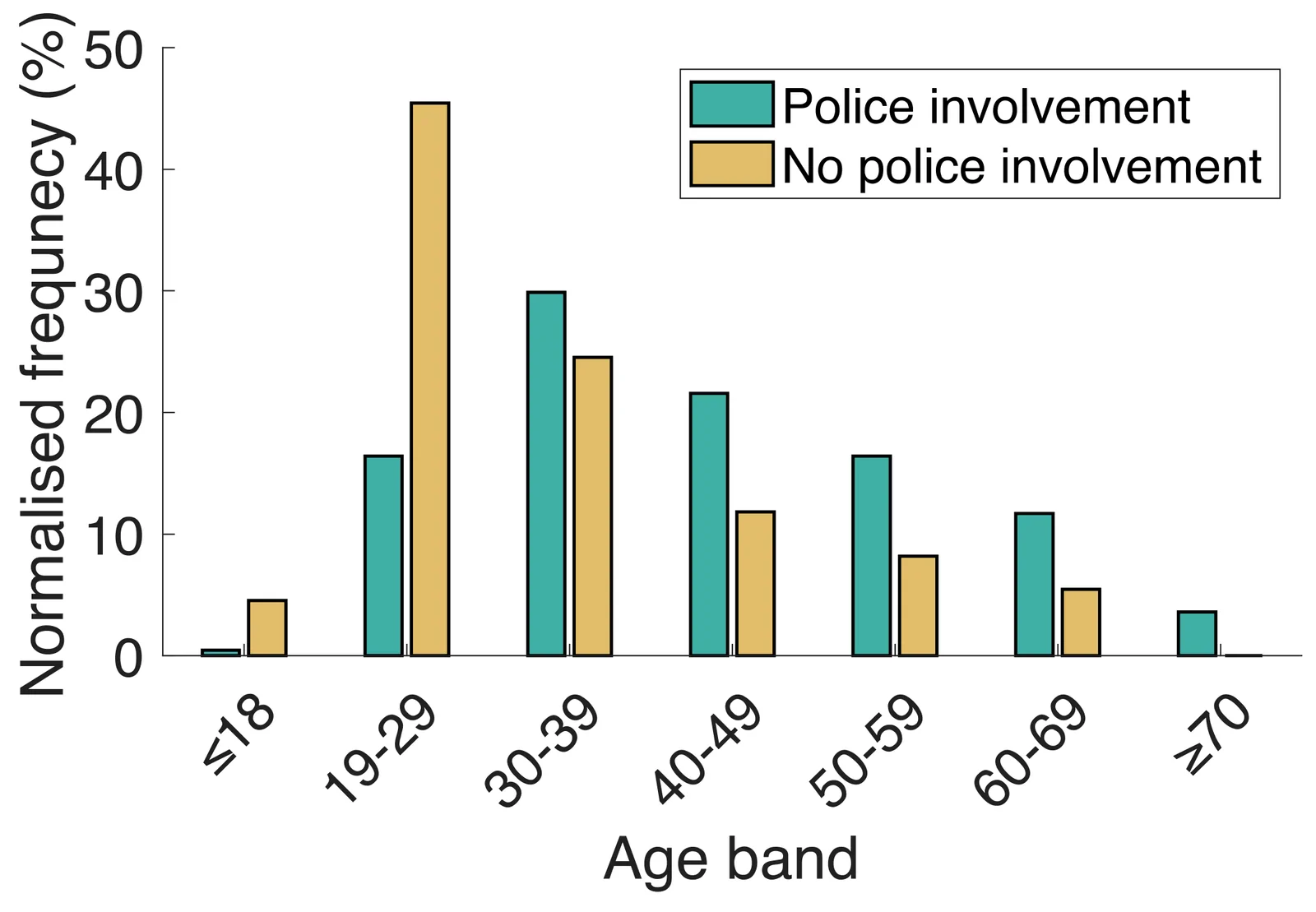
Age Band Distributions of Reported Police Involvement Compared Between Pure Live-Chat and Pure Phoneline Users Concerned About Their Own Behaviour *Note.* Frequencies are normalised against the total number of police-involved individuals (*n* = 459) and those reporting no police involvement (*n* = 116). Median age range of those with prior police involvement 40-49; median for those with no police involvement 19-29 years; Wilcoxon Signed-Rank test: Z = 6.74, *p* < .01.

The age band distributions by reported police involvement of those concerned about their own behaviour are shown graphically in Figure 1. This confirms a significant difference in police involvement by median age, with the age discrepancies particularly evident at the age extremes. None of the 16 users over 69 were without prior police involvement.

**Figure 2:**
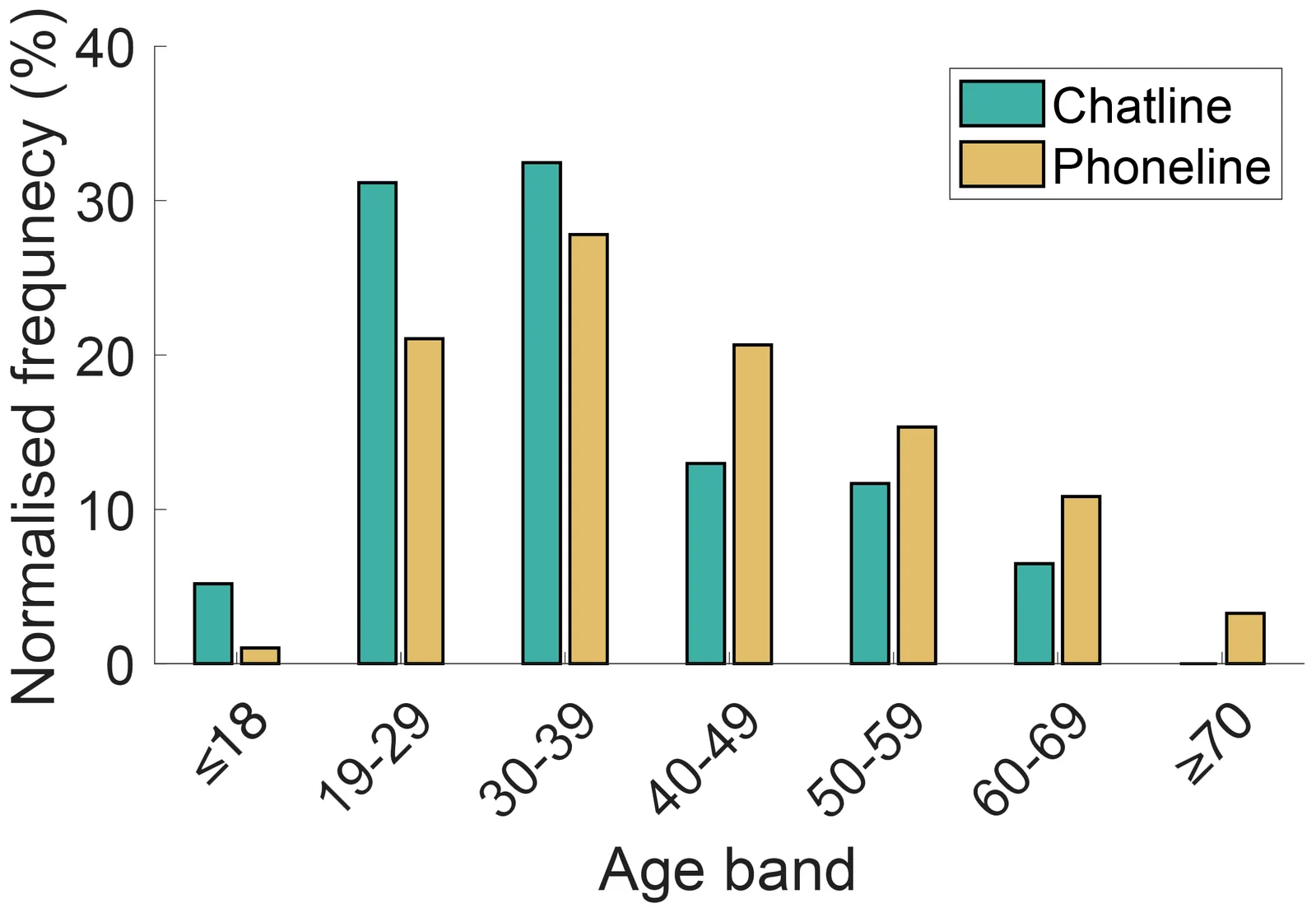
Age Band Distributions of Pure Live-Chat and Pure Phoneline Users Concerned About Their Own Behaviours *Note.* Frequencies are normalised against the respective total number of chatline (*n* = 55) and phoneline (*n* = 489) users. Median age live-chat users: 30-39: phoneline users: 40-49; Wilcoxon Signed-Rank test; *Z* = 3.41, < .01.

A similar examination of age and contact method within the cohort concerned about their own behaviours is shown in Figure 2, confirming the median age of live-chat users was significantly lower than that of phoneline users, and that 14% more live-chat than phonelines users were under 30.

**Figure 3:**
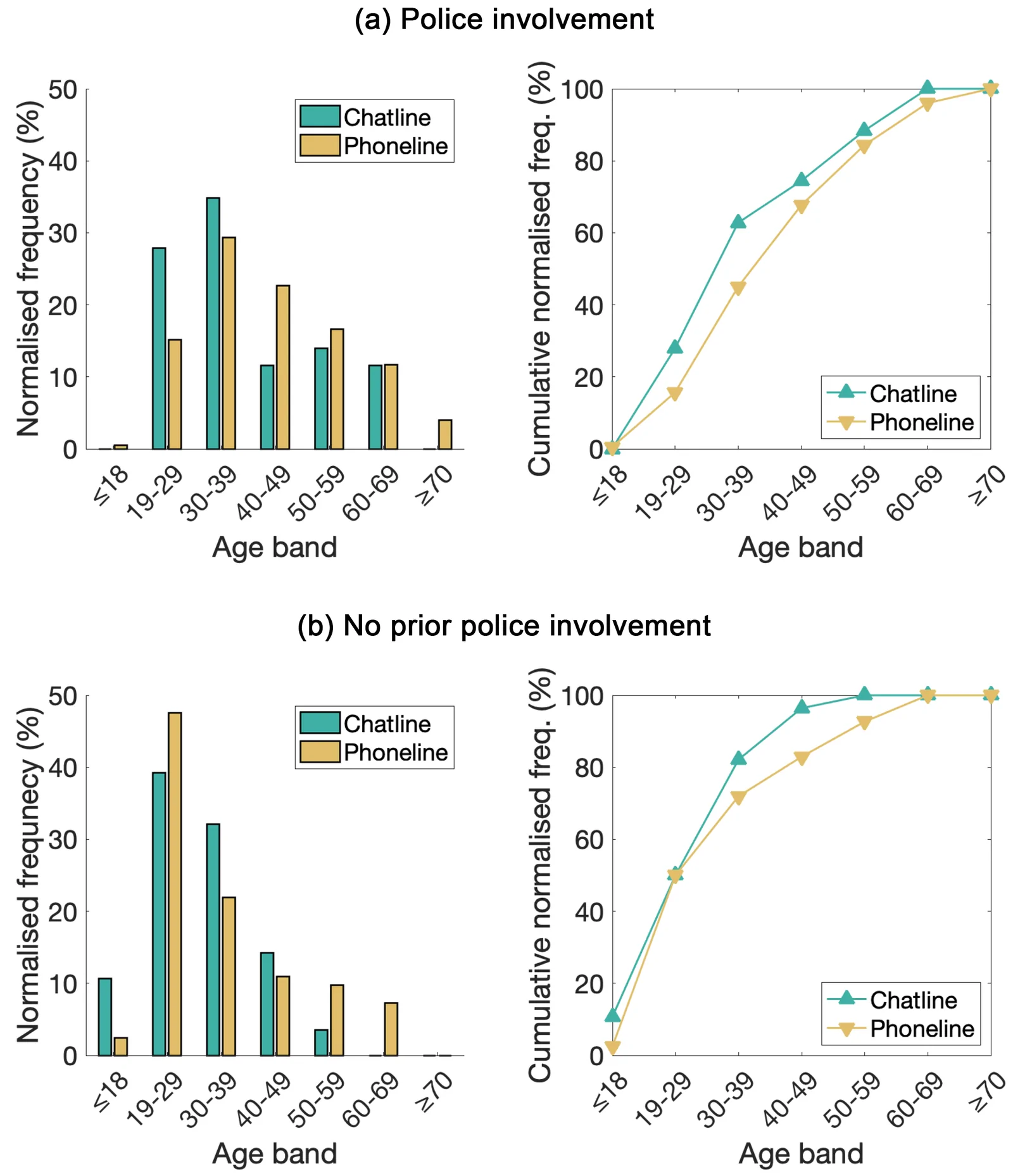
Normalised and Cumulative Frequencies of Age Band Distributions of Self-Concerned Individuals Using the Live-Chat or Phoneline in the (a) Police Involved Subgroup and (b) No Prior Police Involvement Subgroup *Note.* Chatline and phoneline age distributions present similar features in both subgroups (Two-sample Kolmogorov-Smirnov test (a) D = 0.178, *p* = .154, and (b) D = 0.135, *p* = 0.81. Excluding the 22 using *both* live-chat and phone.

Figures 1 and 2 confirm that having no police involvement *and* being a chatline user are both associated with being under 30 (two-sample Kolmogorov-Smirnov test: D = 0.13, *p* = .34) whilst police involvement *and* phoneline use is more common amongst users over 30 (two-sample Kolmogorov-Smirnov test: D = 0.05, *p* = .55). We therefore explored the relationship between all three variables together. Figures 3a and 3b illustrate the relationships between contact medium (live-chat/phoneline), age, and prior police involvement.

Critically, age distributions between chatline and phoneline users are comparable in both subgroups of police involvement, as highlighted by the similarity of the chatline and phoneline curves in the cumulative frequencies of the age distributions. In the police involved subgroup, both chatline and phoneline users have a median age band of 30-39, in contrast to the median age band of 19-29 for the chatline and phoneline users in the no police involvement subgroup. Figure 3 thus confirms user age as the more dominant factor, younger age accounting for both lower likelihood of police involvement and higher likelihood of live-chat than conventional phone use.

### Nature of Service Use: The Qualitative Analyses

#### Single Occasion Live-Chat Users

Data saturation was achieved after analysis of six transcripts, confirmed after no new data categories emerged in analysis of three more scripts. Online Supplement 4 shows all categories with supporting evidence. There, and below, all quotations are reproduced exactly as typed; …. indicates words omitted; a new line indicates a new message within the session; the post-quotation number in bold italics is the unique research ID.

Mean duration of the nine randomly selected single occasion chatters included was 43 minutes (m) and three seconds (s) (range 14m 39s – 84m 27s). They included one woman asking about her partner’s behaviour, one man about a friend and seven men about their own behaviour. Three of the latter seemed to be in the Prochaska and DiClemente (1982) pre-contemplation stage about their problems, two terminating the chat themselves:

e.g. ‘I am not sure why I clicked on to live chat …. I think I’m going to go’ [13m later, he disconnected] **004**

The third chat in this subgroup was ended by the responder; the chatter was not interacting but demanding to be registered as a ‘*none afending pheodophile*’. These brief texts are not considered further here.

‘Worry’ was central to every other chat - about self and/or others, and often about the act of help seeking:

I am worried about myself **003**

I never asked for help because I was too scared **007**

Other negatives were also prominent: ‘stressed’, ‘tiredness’ and ‘loneliness’, feeding into each other.

Reactions to themselves included guilt, shame and, for perpetrators, self-disgust *or* almost out-of-body experiences, including splitting:

I have a problem, I went down a rabbit hole of looking … **003**

What I am talking about is the complete opposite of the true me **006**.

The prospect of change was evident in only the two longer perpetrator chats:

I’m in a loving relationship for the first time … I want to move on **003**

[of court] I want to show I’m getting help … I want to move forward **007**.

In summary, the single chats offered evidence of need and some problem recognition, but little of engagement of the chatter with the responder in any process for change.

#### Multiple Occasion Live-Chat Users

Twenty people used live-chat at least twice. Nine did not meet other inclusion criteria for analysis: one made all three chats on the same day, one connected on separate days but typed nothing and three further transcripts had almost no unprompted speech; four later sought resource information only;

Thus, 11 people initiated at least two live-chats over 24 hours apart, made no phoneline contact and typed some words unprompted or unshaped by the responder; one also used email but never voice contact. As only one was concerned about others’ behaviour, analysis was confined to the self-concerned group, all men. Each is indicated below by a different alphabetical letter; numbers indicate order in the chat series. B excepted, who was also emailing, texting was their sole communication channel. Some typed that they couldn’t talk, explicitly not wanting to hear material out loud.

Number and duration of chats among those included varied, but 2-5 sessions most usual. Rounded to full days, mean time from first to last contact was 40 days, but the range extensive, from 14 chats over 72 days to just two qualifying chats over four days; one man [H] made four contacts over the longest period observed (124 days) but offered only three lines of spontaneous detail in only one, his second. H excepted, everyone thanked the responder, invariably at the end of what proved to be the last chat. Thus, thanks appeared to be a form of farewell. Endings were otherwise unremarkable.

Although some of the verbatim material was rich, little was ideal for qualitative analysis as the responders often asked direct questions, or made recommendations for self-care or interventions. Although in line with the organisation’s policies, this inevitably shaped parts of the chat. Nevertheless, spontaneous concerns came through, and data saturation was achieved after analysis of seven chats (Online Supplement 5).

**Figure 4:**
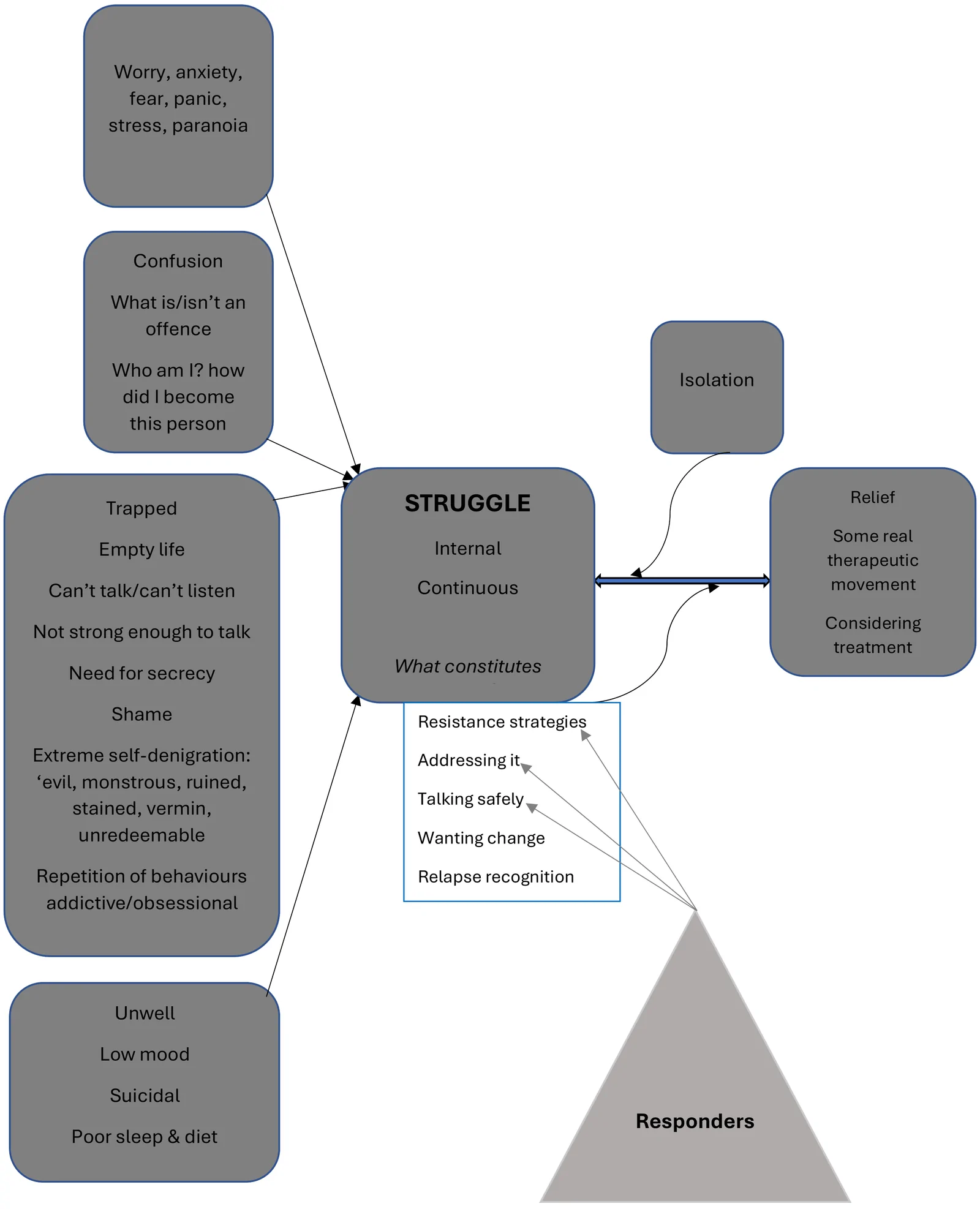
Men Using the Stop It Now Live-Chat Service Because of Their Own Behaviours: A Model of the Concerns of Those Who Chatted on More Than One Occasion on Different Days

A model of the service use emerged (Figure 4).

The core category/concept was of struggle, an internal state, sometimes explicit and generally summarising experiences of a range of emotions between anxiety and panic or paranoia, of confusion, of being trapped in cycles of shame, self-denigration and behavioural repetition and feeling generally low and even suicidal. There was some vision of resolution – as relief from such struggle, perhaps through treatment and real change – but very little experience or expectation of achieving it. Experience of isolation mitigated against resolution, while some strategies, often recommended or reinforced by the responders, appeared to support some positive movement, if only briefly.

Explicit struggle had two elements, sometimes only one of them present. One was wish for clarity on what constitutes crime or harmful behaviour; the other, the more usual, was of managing intense internalised emotional pain about what had been happening. Even the man who only once got beyond typing that he had a problem offered:

Ok, dont why i became this person, it just happened a while ago.

Sometimes, i have had thoughts, which i know isnt right. **H2**

Particularly where there had been no police involvement, confusion and inner conflict were explicitly part of the struggle:

I feel like an awful twisted person but somehow I still get sexual gratification from these thoughts 

Is it possible to be aroused by something and hate yourself for it at the same time? **K1**

Almost everyone had an extremely negative self-perception – self-descriptors included ruined, monster, evil, vermin, scum, the lowest on the planet. Shame was a recurring category, with one man even apologising to the responder for speaking about his problems:

I am sorry you have to hear them [his problems] **D1**

A sense of helpless repetition was intrinsic to this struggle mainly referred to the transgressive behaviours; some offered labels like ‘obsessional compulsive states’ or ‘addiction’, commonly describing trying not to do the acts and failing, then, when most agitated, getting some soothing from the acts, tending to endorse this sense of helpless, before going round the cycle again. Ill health also contributed - poor sleep and nausea were commonly mentioned. Some were despairing, even explicitly suicidal.

Expressions of some relief from the chat occurred, although not always much separated from relapse or deterioration, as for the most prolific chatter (D):

Ill try to think of some small goals I can do, and try to refer myself for talking therapy. **D7**

You guys have helped me a lot, I don’t know where I’d be if it wasn’t for this place. **D10**

I cant move on from this … its just been getting worse and worse. **D14**

## Discussion

No prior study has examined a live-chat service for prevention of sexual harms to children. We found that all live-chat time available was used, with about 75% of those contacting the service doing so in preference to other contact methods. Thus, the service is in both general and specific demand, but availability was only 120 hours over three months.

Comparison of live-chat with phoneline use over similar periods showed that, on average, chatline users were younger and less likely to have been involved with the police. This suggests that live-chat may be reaching people with concerns about sexual abuse of children earlier than other interventions, so has potential for a greater primary prevention role than other helping services.

Although the majority of live-chat contacts were once only, there was some evidence not only for preference of live-chat over other contact measures, but also some service and psychological engagement through live-chat in working on concerns.

### Service Reach

It is perhaps unsurprising that younger people, growing up with chat technology, are more likely to choose it for assistance and this is consistent with a systematic review of research into health promotion chatlines (Brody et al., 2020). Furthermore, detailed examination of people relying solely on live-chat in our study suggested such difficulty in accessing any other help, that there was otherwise nothing between them and harming others. Matteson et al. (2011), albeit researching emotionally neutral use of live chat within library services, found that reasons for preferring live-chat were ‘convenience’ coupled with familiarity with and rejection of other services. The latter is perhaps relevant here as those concerned about their own behaviours described themselves as avoidant of other communication options.

The finding of lower likelihood of police contact in the chat group, as indicated by both ‘bystanders’ and probable/actual perpetrators may simply follow from the fact that younger men had had less time to be arrested. Further, the analyses tend to support this, but the finding is important anyway. It indicates that the chatline may be opening a resource for people not yet under external pressure to seek help, or who find even voice contact difficult and who might otherwise not seek help. Live-chat availability may, thus, create opportunities for primary prevention of harms as well as earlier interruption of harmful pathways.

### Qualities in Service Use

Other research has suggested that discussing child sexual abuse with potential perpetrators could have a positive impact on preventing abuse. This work, however, has focussed on treatment programmes, in all but one study of outcomes, face-to-face treatment (Seto et al., 2024). McKibbin et al. (2017), from semi-structured interviews with 14 young people who had perpetrated harmful sexual behaviour and six treatment-providing workers, found three key opportunities for prevention – the first focussing on helping potential perpetrators manage their use of pornography. Accessing indecent images of children was the commonest concern of those contacting the live-chat service we studied, and there was encouraging anecdotal evidence of live-chat user acceptance of advice on this. The other two opportunities for prevention were attention to own, earlier victim experiences and provision of sexual education. There was certainly some reference to both of these needs even in single-occasion life-chat transcripts.

The abusing or potentially abusing live-chat men studied in detail were in emotional turmoil, sometimes with self-harm or suicide as clear risks. This fitted with the hopelessness and extreme labels they gave themselves. That said, there seems to be an important difference between them and people contacting a live-chat service primarily for suicide-related-behaviour concerns. In a large study of self-reported outcomes after live-chat for suicide prevention, many felt helped and just under half less suicidal (Gould et al., 2021), but shame was not mentioned; in our live-chat group concerned about their own sexual behaviour, shame was common, strongly expressed and may partly account for the low return rates. It is known that, while guilt may have value for resolution, shame is associated with avoidance (Tangney et al., 2011). The transcripts in our study suggested little capacity for self-forgiveness but avoidance at several levels. Even within the relative transience of live-chat work some attention to this may be possible, perhaps enabling a path towards an appropriate sense of guilt, acknowledging wrongdoing together with intent not to repeat it, and working accordingly (Griffin et al., 2016).

We found just three studies of outcome relating to live-chat with more similarities to our study, with young people finding it difficult to access help otherwise and including victims of sexual abuse. Users of a live-chat service for people who had been sexually assaulted were more likely than not to be satisfied with it, satisfaction being associated with perception of responder knowledge and skills (Finn et al., 2011). A more recent study considered how to optimise matching chatter characteristics and responses (Sindahl & van Dolen, 2020). Service users who completed questionnaires reported that longer sessions with high responder density of material were more beneficial than session frequency. That said, these were only chatter *impressions* of benefit. Even within a confidential service this might be researched further, for example by randomising chatters to more open conversational models and more closed advice giving and including number of subsequent calls, quality of exchanges and take up of other services as outcomes. For prevention of child sexual offending, analyses would also have to take account of potential confounding factors such as open versus arrest driven choice to chat, concerns about own behaviour versus concerns about whether or not a specific act constitutes a crime.

### Strengths and Limitations

A limitation of this, and all other services for anonymous users, is that we could not follow through to data on actual prevention. As a first study of a new live-chat service for people concerned about sexual abuse of children, however, added prevention value is implied by the reach to more people without police involvement. Another limitation of studying anonymised services is that the truth of claims cannot be checked, but consistency in descriptions of concerns across contacts by multi-occasion users suggests little conscious dissimulation.

Patterns of service use may change as the service becomes more widely known. Generalisability may also be limited in that such service use differs between cultures; in this context, it is interesting that for this UK based helpline service the largest group of contacts is of people concerned about their own behaviour, whereas in a USA-based sister organisation the largest groups was of people concerned about others (Grant et al., 2019). That said, the youth factor in live-chat use seems a constant across all studies of chat-based services (Kuerbis et al., 2017). No data were available on ethnicity. Although it is recognised that prevention of child sexual abuse is a concern across all communities, there may be particular barriers in this context (e.g. Sawrikar, 2020). This would be an important area for further study.

A strength in the qualitative analyses was that we had verbatim conversation data, a limitation was, therefore, that it was not completely free speech; we compensated for direct questions by relying only on free text sections for coding.

### Conclusions

Our research adds to already published literature as the first study of live-chat service for prevention of child sexual abuse. The service shows promise as all available time was taken, mostly by people who explicitly preferred it to other communication modes. In attracting a younger clientele, less likely to be police involved than conventional phoneline users, it opens new preventive prospects. Qualitative analyses of verbatim transcripts suggested that it may also be preferred by a particularly vulnerable group, otherwise feeling too ashamed to talk. Among men contacting about their own abusive behaviours, few returned for further chats. Men who did were characterised by sense of painful inner struggle with limited vision of resolution in desistence and feeling relief, but there was some evidence of psychological engagement about their difficulties. This suggests that future research should explore strategies to increase engagement.

## Supplementary Materials

The Supplementary Materials include the literature search algorithms (Supplement 1), the full data extraction sheet for the quantitative component of the study (Supplement 2), the full distribution of presenting problems for the years 2018 and 2020 (Supplement 3), categories and their supporting data for the qualitative study of the one-time chatters (Supplement 4) and categories and their supporting data for the multiple occasion chatters (Supplement 5) (for access, see Gregg et al., 2026S).



GreggT.
KalebicN.
MooreJ. W.
NaldrettG.
EdwardsS.
FindlaterD.
TaylorP. J.
 (2026S). Supplementary materials to "First evaluation of a novel live-chat service for preventing child sexual abuse"
[Online Supplements 1-5]. PsychOpen. 10.23668/psycharchives.22449
PMC1361014942788163

## Data Availability

Due to the extremely sensitive nature of the data, in particular the risk that a person may have inadvertently identified him- or her-self in the material obtained, we are unable to make the dataset and transcripts available. We have, however, provided the data collection sheet, a more detailed list of the behaviours of concern and more extensive quotations from the text messages in Supplementary Materials for access, see below).

## References

[sp1_r1] Gregg T. Kalebic N. Moore J. W. Naldrett G. Edwards S. Findlater D. Taylor P. J. (2026 S). Supplementary materials to "First evaluation of a novel live-chat service for preventing child sexual abuse" [Online Supplements 1-5]. PsychOpen. 10.23668/psycharchives.22449 PMC13610149 42788163

